# Autoinflammatory Diseases in Childhood

**DOI:** 10.4274/balkanmedj.galenos.2020.2020.4.82

**Published:** 2020-08-11

**Authors:** Mehmet Yıldız, Fatih Haşlak, Amra Adrovic, Kenan Barut, Özgür Kasapçopur

**Affiliations:** 1 Department of Pediatric Rheumatology, İstanbul University-Cerrahpaşa Cerrahpaşa School of Medicine, İstanbul, Turkey

**Keywords:** Autoinflammatory diseases, childhood, classification, treatment, prognosis

## Abstract

Autoinflammatory diseases are characterized by recurrent fevers and clinical findings of impaired natural immunity and can involve various organ systems. The concept of autoinflammatory disease emerged after the definition of familial Mediterranean fever and tumor necrosis factor receptor-associated periodic syndrome. This new disease group was considered to differ from the standard concept of autoimmune diseases, which is relatively better known in terms of basic features, such as defects in innate immunity and the absence of antibodies. A better understanding has been achieved regarding the genetic and pathogenetic mechanisms of this relatively new disease group over the past 20 years since they were first diagnosed, which have led to some changes in the concept of autoinflammatory diseases. The recent definition classifies autoinflammatory disease to be a wide range of diseases with different clinical features, mainly accompanied by changes in innate immune and rarely in humoral immunity. The spectrum of autoinflammatory diseases is rapidly expanding owing to recent developments in molecular sciences and genetics. This review article discusses the clinical features, classification criteria, treatment options, and long-term prognosis of periodic fever, aphthous stomatitis, pharyngitis, adenitis syndrome, and other common autoinflammatory diseases in the light of current literature.

Autoinflammatory diseases are a group of diseases that occur because of the dysfunction or dysregulation of innate immunity and can cause serious morbidity and mortality by affecting multiple organ systems. This new group differs from the standard concept of autoimmune diseases, which is relatively better known in terms of basic features, such as defects in innate immunity and the absence of antibodies circulating in the bloodstream. The concept of autoinflammatory disease emerged after the definition of familial Mediterranean fever (FMF). Notably, the spectrum of autoinflammatory diseases is rapidly expanding owing to recent developments in molecular sciences and genetics.

## Definition and concept of autoinflammation

Until date, several definitions have been proposed to describe this group of rare diseases (1). The term “autoinflammatory diseases” was first described by the National Institutes of Health as systemic diseases characterized by non-provoking attacks, without high-titrating antibodies and antigen-specific T lymphocytes (2). In 2010, Kastner et al. (3) defined autoinflammatory disease as “clinical disorders marked by abnormally increased inflammation, mediated predominantly by cells and molecules of the innate immune system, with a significant host predisposition”. In 2017, Wekell et al. (4) modified this definition as “autoinflammatory diseases are immunological diseases defined by abnormally increased inflammation, driven by dysregulation of molecules and cells of the innate immune system with a host predisposition as necessary and sufficient criteria, frequently associated with activation of the adaptive immune system and potentially with immune dysfunctions, such as susceptibility to infections, autoimmunity or uncontrolled hyperinflammation.” Finally, in 2018, the Pediatric Rheumatology International Trials Organization (PRINTO) defined autoinflammatory diseases as “clinical disorders caused by defect(s) or dysregulation of the innate immune system, characterized by recurrent or continuous inflammation (elevated acute phase reactants) and the lack of a primary pathogenic role for the adaptive immune system (autoreactive T-cells or autoantibody production)” (1).

The changes in the definition of the disease reflect the expansion of the spectrum and the classification of new diseases under this group. The recent definition of autoinflammatory disease classifies it as a wide range of diseases exhibiting different clinical features, mainly accompanied by changes in innate immune and rarely in humoral immunity. Diseases classified presently under the group of autoinflammatory diseases are summarized in Table 1. This review article discusses the clinical features, classification criteria, treatment options, and long-term prognosis of periodic fever, aphthous stomatitis, pharyngitis, adenitis (PFAPA) syndrome, and other common autoinflammatory diseases.

## Diagnostic approach for patients with suspected autoinflammatory disease

Fever is one of the most common reasons for hospital admission during childhood, with infections being the leading cause of fever in children. The initial step in the evaluation of a child with fever is the definition of fever and its characteristics. It should be kept in mind that parents can subjectively exaggerate the symptoms, and if parents complain regarding recurrent fevers, a fever diary that is filled objectively should be requested. Fever diary is a useful tool in confirming the fever and identifying fever patterns like periodic, recurrent, or prolonged.

**Figure 1:**
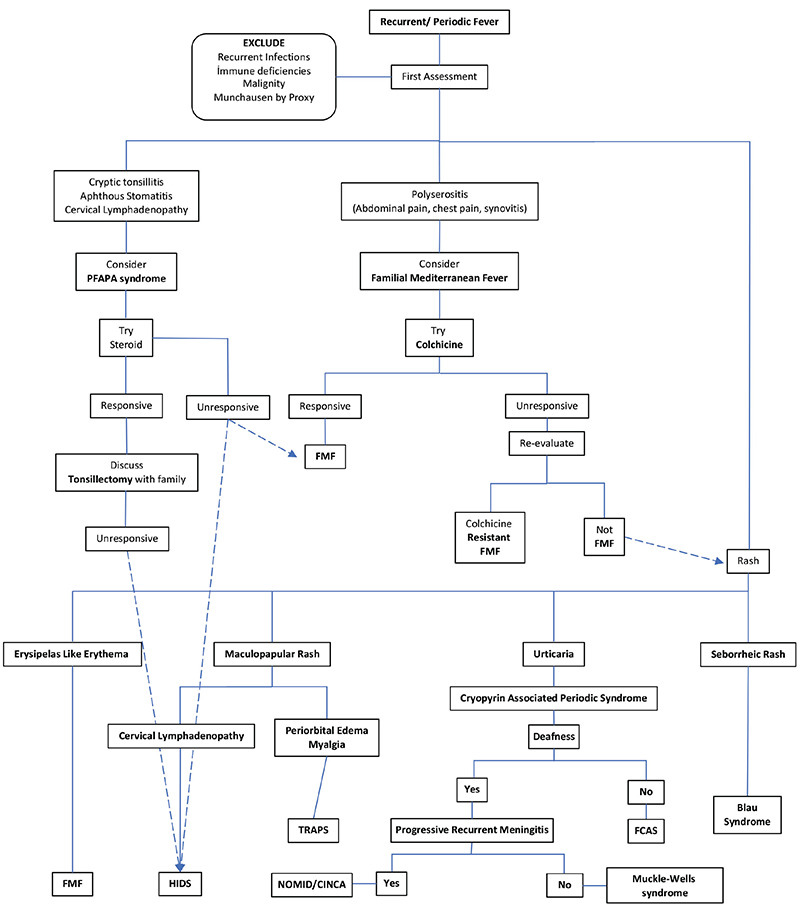
Diagnostic approach algorithm in children with recurrent fever. CINCA: chronic infantile neurological, cutaneous, and articular syndrome, FMF: familial Mediterranean fever HIDS: hyperimmunoglobulin D, NOMID: neonatal-onset multisystem inflammatory disease, PFAPA: periodic fever, aphthous stomatitis, pharyngitis, adenitis syndrome, TRAPS: tumor necrosis factor receptor-associated periodic syndrome

In the case of recurrent or periodic fever, a detailed medical history of the patient and family should be obtained, and cautious physical examination should be performed. Nonetheless, considering the rarity of autoinflammatory diseases, other possible reasons for periodic or recurrent fever should be considered, such as recurrent infections, malignancies, immunodeficiencies like cyclic neutropenia. Moreover, fever can be a sign of Munchausen syndrome by proxy, and accordingly, physicians should be vigilant regarding every child with recurrent fever. While developmental delay, growth restriction, and history of hospitalization for severe infections primarily suggest possible immunodeficiency, night sweats, night pains, weight loss, generalized lymphadenopathy, or hepatosplenomegaly may indicate malignancies. On the other hand, indications suggestive of autoinflammatory diseases are normal growth and development patterns, asymptomatic between episodes, positive family history, and history of similar episodes. A diagnostic approach algorithm is presented in Figure 1.

## Periodic fever, aphthous stomatitis, pharyngitis, adenitis syndrome

PFAPA syndrome is one of the most common periodic fever syndromes observed during childhood (5). However, the etiology and genetic basis of this unique disease is still unclear (6). Literature reveals a common misuse of nomenclature of diseases with recurrent or periodic fever. Most authors classify the autoinflammatory diseases under an umbrella of periodic fever, although most of these diseases have recurrent fever rather than periodic fever. The most crucial exception is PFAPA syndrome. The episodes of PFAPA syndrome typically have a perfect periodicity that allows families to predict the occurrence of the next episode.

The genetic background of this syndrome is still a critical topic of discussion and research. Although the literature reports a high rate of positive family history in patients with PFAPA syndrome suggestive of possible genetic transmission, Di Gioia et al. (7) could not determine a causative gene by conducting whole-exome sequencing studies, and they suggested that PFAPA is related to the oligogenic or complex inheritance of various genetic and non-genetic factors (8,9,10). The relationship between PFAPA syndrome and other autoinflammatory disease genes is another topic of interest. Patients with underlying pathogenic variants of other autoinflammatory diseases tend to have an atypical presentation of PFAPA syndrome (11,12). Some studies in the literature have reported an increased frequency of Mediterranean Fever (*MEFV*) gene mutations in patients with PFAPA syndrome. The frequency of the *MEFV* mutations in patients with PFAPA syndrome is reported to be 8%-66% (8,12,13,14,15). It has been reported that patients with PFAPA having *MEFV* mutations have shorter episodes, longer inter-episodic intervals, and lower corticosteroid dose requirement for the disease attacks cessation (16,17,18). In contrast, Pehlivan et al. (15) observed that patients with PFAPA syndrome having underlying *MEFV* mutations have a severe clinical course.

Although sporadic adult cases have been reported, the disease typically occurs in children younger than 5 years of age (5). Periodic fever is the most prominent finding of the disease. The fever is typically accompanied with aphthous stomatitis, pharyngitis, and adenitis. The episodes often end within a week and recur every 2-8 weeks (11). The early episodes typically exhibit perfect periodicity, enabling the family to predict the next occurrence. The periodicity of the episodes typically disappears during the disease course, the attack-free intervals become longer, and finally, the episodes cease 3-5 years after the onset (5,11). Notably, no developmental and growth delays occur in children with PFAPA syndrome. Fever in PFAPA syndrome can be as high as 39-40°C, typically unresponsive to antipyretics. Despite the high fever, children do not appear ill, and this is a useful clue for diagnosing PFAPA. Exudative tonsillopharyngitis or cryptic tonsillitis is another crucial finding of the disease and often occurs along with aphthous stomatitis. The oral findings of the disease typically disappear rapidly within a day after a single dose of steroids. In addition, cervical lymphadenitis is commonly observed during the disease attacks, and this is most often bilateral and tender (5). Because enlarged cervical lymphadenitis is one of the clinical findings of hyperimmunoglobulin D syndrome (HIDS)—another autoinflammatory disease—it is sometimes misdiagnosed as PFAPA syndrome. Therefore, HIDS should be considered in patients with PFAPA unresponsive to tonsillectomy. Nevertheless, abdominal pain, arthralgia, myalgia, and headache are rarely reported during disease episodes (11).

PFAPA is diagnosed based on clinical findings. The most commonly used diagnostic criteria for PFAPA syndrome are modified Marshall’s criteria (19). According to these criteria, diagnosis is based on regularly recurring fever with an early onset (<5 years), absence of cyclic neutropenia, being completely asymptomatic between episodes, normal development and growth, and at least one of the following three criteria: aphthous stomatitis, cervical lymphadenitis, and pharyngitis (19). In 2018, Vanoni et al. (20) proposed a new set of classification criteria for PFAPA syndrome. The sensitivity and specificity of this new set of criteria were reported as 89.7% and 69.5% by Adrovic et al. (21), and they suggested that these newly proposed criteria are insufficient in distinguishing FMF from PFAPA syndrome, especially in regions endemic for FMF. Eurofever/PRINTO suggested new sets of classification criteria for PFAPA syndrome with a sensitivity of 97% and specificity of 93% (22). The diagnostic or classification criteria for PFAPA are presented in Table 2.

Although PFAPA syndrome is a benign condition and typically resolves spontaneously after 5 years of age, the recurrent episodes can exhaust the families and children, forcing them to seek treatment for the disease. Nonetheless, only few drugs have been proven effective in treating PFAPA syndrome. Notably, colchicine, cimetidine, vitamin D, anti-interleukin 1 (IL-1) therapy, and tonsillectomy are the recommended treatment options for the disease (23). Although steroids have been noted to be highly effective in terminating the episodes, it is shown that using steroids shortens the intervals between fever attacks (23). Considering the possible side effects of steroids, it should not be used routinely in all the episodes. Notably, being responsive to steroids can be accepted as a clue for diagnosis of PFAPA syndrome and trying the steroid treatment in patients with PFAPA syndrome suspicion may be logical. Colchicine has been shown to be effective in reducing the frequency of the episodes, with a more prominent effect in patients with *MEFV* mutation in some studies (11,14,15). Therefore, colchicine should be considered before performing tonsillectomy in a certain percent of PFAPA patients, especially those from regions endemic for FMF. Tonsillectomy is another treatment option for PFAPA syndrome. The efficacy of tonsillectomy is reported to be 92% in the literature (11). Because PFAPA syndrome is a benign condition, tonsillectomy should be discussed in detail with the family during decision-making.

## Familial Mediterranean Fever

FMF is the most common hereditary autoinflammatory disease with an autosomal recessive inheritance pattern, characterized by recurrent fever attacks and polyserositis (24,25). The disease occurs most frequently in communities living in the Mediterranean basin, with the highest prevalence reported among Sephardic Jews, Armenians, Turks, and Arabs (26).

*MEFV* encoding pyrin protein is the causative gene of the disease (27). Although the classical inheritance pattern of the disease is autosomal recessive, a vertical inheritance pattern resembling pseudo-dominant inheritance has also been reported in some families from regions with a high carrier frequency of MEFV variants (28). The carrier frequency rate was reported to be approximately 1/6 among Sephardic Jewish, 1/5 among Armenian, and 1/8-1/5 among Turkish people. Thus far, 374 variants of this gene have been identified (https://infevers.umai-montpellier.fr/web/) (29). The most common disease-causing mutations are M694V, V726A, M694I, and M680I, located in the exon 10 (30). It is well-known that, unlike other diseases with autosomal recessive inheritance, the classical FMF phenotype can be noted in patients carrying only one MEFV mutation and even no MEFV mutations. Therefore, the role of gene analysis in diagnosis is limited, and it is typically used for predicting the prognosis or course of the disease. Notably, patients with homozygous M694V mutations have been reported to have severe disease and an increased risk of amyloidosis (24).

In most patients, the disease occurs during childhood, with the first disease episode typically occurring during the first 10 years of life (31). The mean age of the disease onset was reported to be 3-9 years (24). It is characterized by fever and abdominal pain episodes resembling an acute abdomen. Contrary to the nomenclature that named these diseases as periodic fever syndromes, it is more accurate to define the disease episodes as recurrent instead of periodic (24). Nonetheless, the episodes typically resolve within 72 h. Although an apparent prodromal period has been reported in adult patients, episodes in children often start suddenly without any prodromal findings, as well as end spontaneously (25,32). This difference in symptom presentation could be because of the inability of young children to express their pre-attack symptoms, and the prodromal period could be an overlooked finding of the disease in children (25). Despite various types of disease episodes, the most common clinical phenotype is the coexistence of fever, abdominal pain, and articular findings.

**Figure 2:**
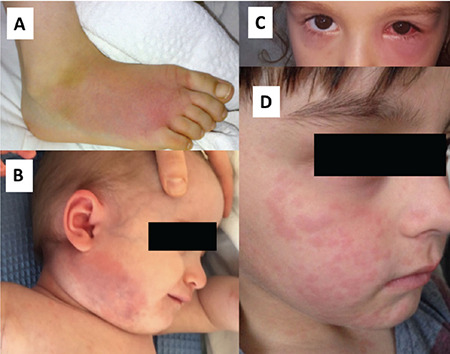
**A-D.** Skin findings of the autoinflammatory diseases. A) Erysipelas-like erythema, B) cervical lymphadenopathy in patients with hyperimmunoglobulin D syndrome, C) periorbital edema, D) urticarial rash in patients with familial cold autoinflammatory syndrome.

Notably, fever is one of the most commonly reported findings of the disease (33). Even though it can be the only finding, it is often accompanied by at least one of the other findings. Another crucial finding of the disease is severe abdominal pain resembling an acute abdomen (34). Because abdominal pain is very severe, most patients are misdiagnosed as acute appendicitis during attacks, and an appendectomy is performed before the FMF is diagnosed (34). The reason for the abdominal pain is aseptic serositis, which is also responsible for chest pain that can be caused by pericarditis or pleuritis (34). Another common finding of the disease is articular involvement, and it could be the only presentation of the disease. The typical articular involvement of the disease causes non-erosive, non-migratory mono- or oligoarthritis of lower extremities that resolves spontaneously within a week. Chronic arthritis has been reported in 2%-5% of patients (24). An erythematous rash can be observed over the involved joint, and this unique sign is termed as “red arthritis” (24). In addition, seronegative sacroiliitis has been reported in patients with FMF (33,35). The most common skin finding in FMF is erysipelas-like erythema that is typically noted on the dorsum of the foot (24) (Figure 2A). In addition, orchitis and aseptic meningitis have been reported in FMF (24). Myalgia is another crucial symptom frequently noted in patients with FMF (24). Apart from being the presenting symptom of FMF, myalgia can also be a sign of protracted febrile myalgia. It is a rare vasculitic manifestation of the disease, which is characterized by symmetric severe muscle pain, tenderness, elevated acute phase reactants, normal muscle enzyme levels, excellent response to steroids, and absence of articular findings (36).

Apart from protracted febrile myalgia, the frequency of other vasculitides, such as Henoch Schoenlein purpura, polyarthritis nodosa, Behcet’s syndrome, and even some other inflammatory diseases like juvenile idiopathic arthritis, psoriasis, and inflammatory bowel diseases are observed to be increased in patients with FMF (37,38).

FMF is diagnosed based on clinical findings. Mutation testing is not required in all suspected patients. As mentioned earlier, knowing the genotypes of *MEFV* mutations provides an insight into the course and prognosis of the disease. Because FMF represents the oldest autoinflammatory disease, several classifications and diagnostic criteria have been suggested. The most commonly known and used ones are Tel Hashomer and Yalcinkaya-Ozen criteria (39,40). According to Yalcinkaya-Ozen criteria, at least two of the following five criteria are required for diagnosis: fever, abdominal pain, chest pain, oligoarthritis, and positive family history of FMF. Moreover, these clinical symptoms should be observed at least three times and should last between 6-72 h to be considered as a criterion (39). Recently, two new sets of classification criteria (combined and only clinical) were proposed by PRINTO (Table 3) (22). The main difference between the new criteria and the earlier one is the inclusion of patient genotype. Sag et al. (41) reported that this new set of criteria is more sensitive (96%) and less specific (73.1%) than Tel Hashomer and Yalcinkaya-Ozen criteria.

The primary aim of FMF treatment is to prevent amyloidosis—the most critical complication of the disease. Notably, colchicine is the mainstay of FMF treatment. Colchicine was first shown to be effective in FMF by Ozkan et al. (42) and then Goldfinger (43). The recommended dose is 0.5 mg (or 1 mg) for children under 5 years of age, 0.5-1 mg for children between 5 and 10 years of age, and 1-1.5 mg for children older than 10 years of age. The maximum dose of colchicine for children is 2 mg per day. In the case of uncontrolled episodes with 2 mg colchicine per day, drug compliance should be assessed. Other treatment options like biologic agents should be considered in patients unresponsive to the maximum tolerable dose of colchicine to prevent amyloidosis. Although several definitions of colchicine resistant FMF exist in the literature, according to the most used one, colchicine resistance is defined as three or more attacks in 6 months or six or more attacks per year despite the appropriate drug compliance (44). In our daily practice, we have observed that some patients benefit from changing the brand of colchicine in the case of colchicine resistance, and this approach sometimes helps to get rid of the colchicine side effects like diarrhea. A recent study by Emmungil et al. (45) revealed that adult patients who are not responsive to domestic coated colchicine tablets could benefit from compressed colchicine preparations. Although not much evidence exists for this approach in children, it may be cost-effective and logical to try another brand of colchicine before using the biologic agents. In the case of colchicine resistant disease, anti-IL-1 agents like anakinra and canakinumab are recommended (46,47).

## Hyperimmunoglobulin D syndrome or mevalonate kinase deficiency

HIDS is an autosomal recessive autoinflammatory disease. It is caused by mutations in the *MVK* gene that encodes mevalonate kinase, an enzyme that plays a crucial role in cholesterol biosynthesis (48). Of the approximately 100 described mutations of the *MVK* gene, V377I has been reported to be the most common variant (49). MVK mutations lead to a decrease in the downstream product of the mevalonate kinase pathway, causing IL-1 β secretion with resultant autoinflammation (50).

Disease severity highly correlates with the remaining enzymatic function (51). The *MVK* enzyme activity in *MVK*-HIDS is reduced to 1%-8% of normal, whereas in mevalonic aciduria (MA), it is below 1% (52). *MVK*-HIDS is a milder condition, characterized by febrile episodes, combined with non-specific skin rashes, cervical lymphadenitis, arthritis or arthralgia, and severe gastrointestinal complaints, sometimes provoked by vaccination or infections (Figure 2B) (53). Episodes typically last 3-7 days and recur every 4-6 weeks (54). The frequency and severity of episodes decrease with age (55). Contrary to the nomenclature of the disease, 28% of patients do not have elevated immunoglobulin D levels (48). On the other hand, MA is more severe, characterized by episodes similar to those in mevalonate kinase deficiency-HIDS, but with a chronic disease course (56). In addition, mental retardation, dysmorphic features, and failure to thrive can be observed rarely in MA (57).

According to recently proposed classification criteria, the presence of pathogenic (or likely pathogenic) *MVK* variants (homozygous or in trans compound heterozygous) and at least one of the following symptoms are needed for diagnosis: gastrointestinal symptoms, cervical lymphadenitis, and aphthous stomatitis (22) (Table 4). The sensitivity and specificity of the new criteria were reported to be 98% and 100%, respectively (22).

Corticosteroids have been shown to be effective in reducing symptoms, particularly during episodes (58). If steroids are unable to suppress the flares, treatment with a biological agent such as anti-IL-1 agents or anti-TNF agents should be considered (59,60).

## Tumor necrosis factor receptor-associated periodic syndrome

TRAPS is an autoinflammatory disease with periodic fever, musculoskeletal symptoms, skin changes, and eye findings (61). Although its exact prevalence is unknown, it is considered the most common autoinflammatory disease with an autosomal dominant inheritance pattern (34,62).

The causative gene of the disease is *TNF Receptor Super Family 1A (TNFRSF1A)* that is located on chromosome 12 (63,64,65). Most of the disease-related genes are located on exons 2, 3, and 4, and approximately 167 sequence variants have been identified thus far (https://infevers.umai-montpellier.fr/web/) (29). *TNFRSF1A* mutations are divided into the following two groups: structural and non-structural mutations. Structural mutations, especially in which cysteine bonds are affected, are reported to be associated with more severe disease and increased risk of amyloidosis (61,62,63,66). The most common mutations detected in patients with TRAPS are T50M and T50K related to hydrogen bonds (67). Mutations wherein the cysteine bonds are affected, such as C29S, C29Y, C29F, C29G, C29R, C30F, C30Y, C30S, C30R, C33G, and C33Y, and wherein the proline residues are affected, such as P46L, L67P, S86P, and R92P, are other significant examples of structural mutations (61,62,67). Non-structural mutations, such as R92Q and P46L, are frequently noted in healthy individuals. Hence, their pathogenic significance is still controversial (6,61,67). Patients with these mutations can develop clinical findings consistent with TRAPS but have a milder clinical course than patients with structural mutations (6,47).

TRAPS can affect most organ systems and exhibit highly variable clinical findings. The most prominent finding of the disease is recurrent fever attacks that typically last 1-3 weeks and recur every 6 weeks (68). The disease duration is often longer than the other autoinflammatory diseases, and a persistent inflammatory course with intermittent flares is reported in some patients (34). Episodes of the disease typically start with muscle cramps or myalgia, followed by fever, skin rash, arthralgia, arthritis, and findings of the eye involvement. The most commonly reported skin rash is a migratory, centripetal, and erythematous rash (69). Rashes are typically observed on the muscle group exhibiting myalgia. Migratory myalgia is one of the most crucial clues for the disease and is reported to be detected in most patients (62). In addition, ocular findings like ocular pain, conjunctivitis, optic neuritis, and uveitis can be observed. Periorbital edema is pathognomonic for TRAPS, and should, therefore, be investigated in every patient with periodic fever (61,62) (Figure 2C). Polyserositis is another well-defined finding of the disease and can be the only disease presentation (70,71,72). Severe abdominal and thoracic pains could be present (70,71). Furthermore, per the literature, recurrent pericarditis was reported as the only finding in some patients with TRAPS (72). In addition, non-erosive mono- or oligoarthritis, sacroiliitis, headache, scrotal pain, urethral stricture, and behavioral changes can be observed (62,73,74).

Like the other autoinflammatory disease, the most significant complication of the disease is amyloidosis. Reportedly, the risk of amyloidosis increases, especially in the presence of structural mutations that affect cysteine residues (75).

Although various classification criteria for TRAPS have been proposed, the most recent one is the Eurofever/PRINTO criteria (22) (Table 5). The sensitivity and specificity of the new criteria were reported to be 95% and 99%, respectively (22).

The primary aim of treatment is to urgently control the disease activity to resolve the illness, prevent treatment-related damages, and increase the quality of life of patients (47). Although nonsteroidal anti-inflammatory drugs (NSAIDs) have some symptomatic benefits in most patients, they often fail to cease the attacks (34). Steroids are generally successful in terminating attacks when used at a dose of 0.5-1 mg/kg; however, in most patients, their efficacy decreases over time, and their long-term use is not recommended owing to their serious side effects (34,47). Moreover, steroids are reported not to prevent amyloidosis, and often disease relapses after cessation (76). Ter Haar et al. (76) reported that patients with R92Q mutation respond better to NSAIDs and colchicine. In light of this information, it may be a logical strategy to try NSAIDs and steroids in patients with mild clinical findings, if needed, for a short period. Etanercept has been noted to be effective in preventing attacks and reducing steroid dose in some patients with TRAPS (62,77). The efficacy of etanercept treatment decreases over time, and generally, another biological agent is needed in the process (76). Other anti-TNF agents, such as infliximab and adalimumab, have been noted to be ineffective and can paradoxically increase attacks (76). Another group of drugs used in the treatment of TRAPS is anti-IL-1 agents. Anakinra and canakinumab have been effective in both stopping and preventing attacks (78,79).

## Cryopyrin associated periodic syndrome

Cryopyrin associated periodic syndrome (CAPS) is an autoinflammatory disease with the following three distinct subtypes: familial cold autoinflammatory syndrome (FCAS), Muckle-Wells syndrome (MWS), and chronic infantile neurological, cutaneous, and articular (CINCA) or neonatal-onset multisystem inflammatory disease (NOMID) (80). These three subtypes are considered the spectrum of a disease rather than separate entities. Some authors have suggested that the name of the disease should be changed to “*NLRP3* associated autoinflammatory diseases,” which reflects the responsible gene and covers all the three subtypes (6,80).

Gain-of-function mutations in *NLRP3 (CIAS1)* gene encoding cryopyrin protein are responsible for CAPS (81).

All three subtypes of the disease reflect the different levels of disease severity and have both common and unique findings. Fever, flu-like symptoms, rashes, as well as ocular and central nervous system involvement can be observed in all three subtypes (80,82). Disease episodes typically start with fever, fatigue, and flu-like nonspecific findings. Notably, the episodes typically end within a day in FCAS, whereas it can continue for three or more days in MWS and CINCA/NOMID. Furthermore, in CINCA/NOMID, a persistent inflammatory course with intermittent flares is reported in some patients (80). Skin rashes are often the first and prominent finding of the disease. Although urticaria is the most common skin finding, an erythematous rash, edematous papule, and plaque-like skin changes can be observed, too (Figure 2D). Histopathological evaluation of the urticaria-like rashes revealing the absence of mast cells helps in confirming these rashes as not real urticaria (83).

Musculoskeletal findings are another significant sign of the disease. Even though a pain in the extremities, myalgia, and arthralgia can be observed in all forms of the disease, arthritis is often seen in MWS and CINCA/NOMID and can be erosive in patients with CINCA/NOMID (82,84). Rarely, patients can develop bone deformities because of the uncontrolled growth of the patella, articular cartilage, or epiphysis of long bones, and this abnormal bone formation is unresponsive to anti-IL-1 treatment unlike other symptoms of the disease (82).

Progressive hearing loss is one of the main findings in patients with MWS and CINCA/NOMID but can be prevented and even partially regress with timely treatment (34,82). Like the other symptoms of the disease, the severity of the ocular findings increases from FCAS to CINCA/NOMID. Uveitis, papilledema, and optic atrophy have been reported in CINCA/NOMID and rarely in MWS (80,82,85,86).

Central nervous system involvement is a crucial cause of morbidity. Kilic et al. (87) reported that central nervous system involvement was detected in 50% of their patients with CAPS. The mildest form of central nervous system involvement is a headache, and it can be observed in all subtypes of the disease. Aseptic meningitis and increased intracranial pressure are among the common findings in patients with CINCA/NOMID (82). Notably, chronic leptomeningeal inflammation can cause adhesions and hydrocephalus (87). Developmental retardation, seizures, stroke, and vascular occlusions are the other central nervous system findings of the disease (82).

**Figure 3:**
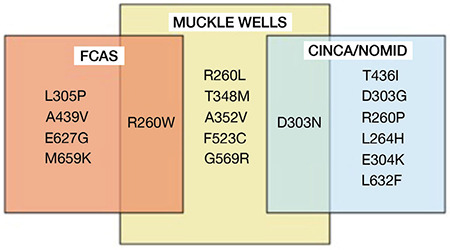
Pathogenic *NLRP3* gene variants according to Infevers database (29) and related phenotypes

Thus far, 230 sequence variants have been identified in this gene (https://infevers.umai-montpellier.fr/web/) (29). Most of the disease-causing mutations are located in exon 3 of the gene. Figure 3 presents the mutations classified as pathogenic by Infevers database and the related phenotypes. Somatic mosaicism was reported in some patients with clinical findings consistent with CAPS, but none of the mutations were observed (88,89). Therefore, it should be kept in mind that the absence of the NLRP3 gene mutation in patients with a strong suspicion of CAPS will not exclude the diagnosis, and patients should be evaluated regarding somatic mosaicism, if necessary.

The most recently proposed classification criteria for CAPS are the Eurofever/PRINTO criteria (22) (Table 6). In that study, researchers suggested two sets of criteria. Notably, one set consists of only clinical criteria that is a modified version of the classification criteria by Kuemmerle-Deschner et al. (90), whereas the other has genetic and clinical issues. The sensitivity and specificity of these criteria were both reported to be 100% (22).

Like in all the other autoinflammatory diseases, the primary purpose of treatment is to urgently control the disease activity, to resolve the illness, prevent treatment-related damages, and increase the quality of life of patients (47).

Because an increase in IL-1 cytokine level plays a crucial role in the pathogenesis of the disease, the appropriate treatment approach is to antagonize the effect of IL-1 (47,80,82). Various studies have observed anakinra, canakinumab, and rilonacept to be effective in patients with CAPS (82,91,92,93,94).

In conclusion, the number of autoinflammatory diseases is increasing every day. Therefore, these diseases should be considered in the presence of skin and musculoskeletal system findings with accompanying periodic or recurrent fever during childhood. The diagnostic algorithm for suspected autoinflammatory diseases according to clinical findings is summarized in Figure 1. Despite the variable clinical findings of these diseases, the detection of pathogenic mutations strengthens the diagnostic and treatment approaches (95). Nevertheless, because most of these diseases are associated with the IL-1 pathway, anti-IL-1 treatment is the cornerstone in most of them.

## Figures and Tables

**Table 1:**
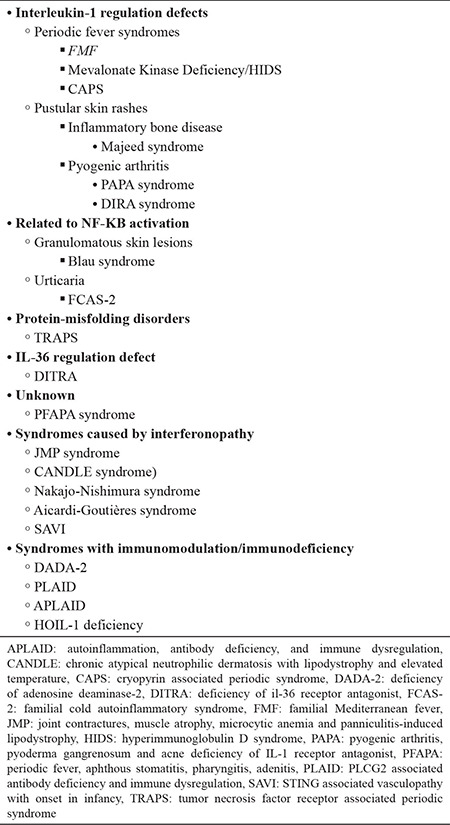
Classification of the autoinflammatory diseases based on the pathogenesis

**Table 2:**
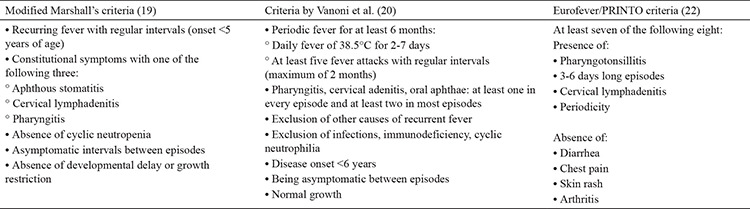
The proposed classification criteria for periodic fever, aphthous stomatitis, pharyngitis, adenitis syndrome

**Table 3:**
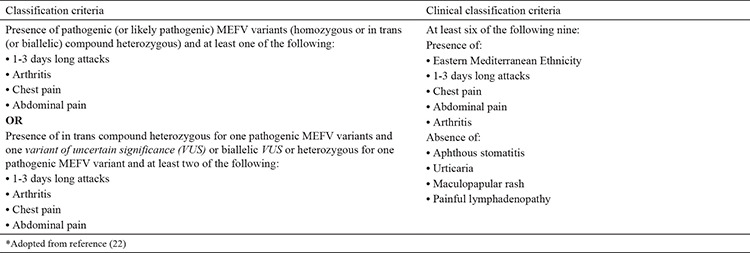
Eurofever/Pediatric Rheumatology International Trials Organization classification criteria for *Familial Mediterranean Fever* (22)

**Table 4:**
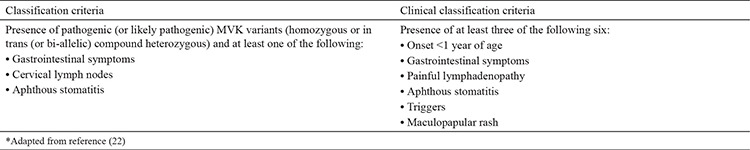
Eurofever/Pediatric Rheumatology International Trials Organization Hyperimmunoglobulin D Syndrome classification criteria (22)

**Table 5:**
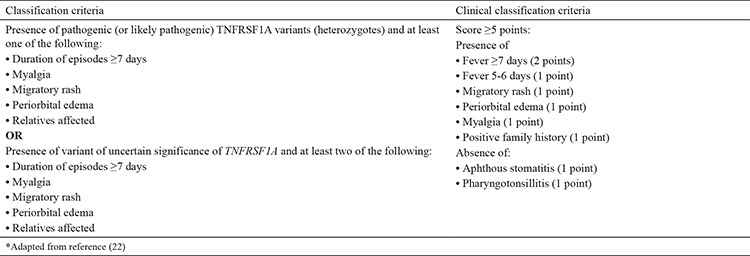
Eurofever/Pediatric Rheumatology International Trials Organization tumor necrosis factor receptor-associated periodic syndrome classification criteria (22)

**Table 6:**
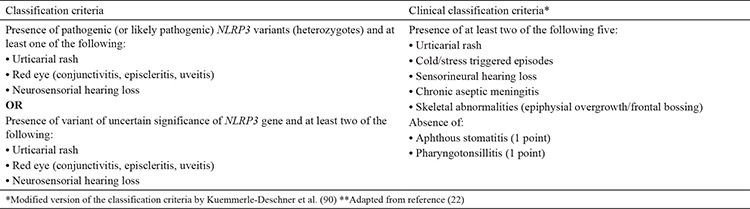
Eurofever/Pediatric Rheumatology International Trials Organization classification criteria cryopyrin associated periodic syndrome (22)
